# The Role of Double-Diffusion Convection and Induced Magnetic Field on Peristaltic Pumping of a Johnson–Segalman Nanofluid in a Non-Uniform Channel

**DOI:** 10.3390/nano12071051

**Published:** 2022-03-23

**Authors:** Yasir Khan, Safia Akram, Maria Athar, Khalid Saeed, Taseer Muhammad, Anwar Hussain, Muhammad Imran, H. A. Alsulaimani

**Affiliations:** 1 Department of Mathematics, University of Hafr Al-Batin, Hafr Al-Batin 31991, Saudi Arabia; yasirmath@yahoo.com (Y.K.); drmathscience51@gmail.com (H.A.A.); 2 MCS, National University of Sciences and Technology, Islamabad 44000, Pakistan; drsafiaakram@mcs.edu.pk (S.A.); m.imran@mcs.edu.pk (M.I.); 3 Department of Mathematics, National University of Modern Languages, Islamabad 44000, Pakistan; maria.athar@numl.edu.pk; 4 Department of Mathematics, Comsats University, Islamabad 45550, Pakistan; sp19-pmt-002@isbstudent.comsats.edu.pk; 5 Department of Mathematics, College of Sciences, King Khalid University, Abha 61413, Saudi Arabia; 6 Department of Mechanical Engineering, School of Mechanical and Manufacturing Engineering, National University of Sciences and Technology, Islamabad 44000, Pakistan; anwar.hussain@smme.nust.edu.pk

**Keywords:** double-diffusion convection, nanofluids, peristaltic flow, induced magnetic field, non-uniform channel, Johnson–Segalman fluid model

## Abstract

The peristaltic propulsion of a Johnson–Segalman nanofluid under the dependency of a double-diffusion convection and induced magnetic field was investigated in this study. On the premise of continuity, linear momentum, solute concentration, thermal energy, and nanoparticle concentration, a flow issue was proposed. The lubrication methodology was used to carry out mathematical modelling. Numerical techniques were used to solve the corresponding highly nonlinear partial differential equations. The exact solution of concentration, temperature, and nanoparticle were computed. The manifestations of all relevant constraints were theoretically and graphically evaluated. The current study develops a theoretical model that can predict how various parameters affect the characteristics of blood-like fluid flows.

## 1. Introduction

Choi [1] introduced the terminology nanofluid which is a heterogeneous mixture of nanoparticles (1–100 nm) into the base fluid. The nanoparticles are generally made up of metal and their oxides. Nanofluid offers many ways to improve the performance of heat transmission in base liquid. Nanoparticles boost thermal conduction of base fluid which consequently increases the heat transfer rate of fluid, and these characteristics make them useful for many industrial applications. The dispersion of nanosized particles has advantages over millimeter- and micrometer-sized particles as bigger sized particles get suspended in the base fluid easily. The nanoparticles, when mixed properly behave similar to a base fluid and avoid the chances of agglomeration and clogging during flow in the channel. In comparison to the conventional ways of heat transfer intensification, nanotechnology offers many advantages such as predominant Brownian motion of particles with high dispersion stability; high specific surface area which offers higher heat transfer rate; reduction in clogging during flow; and adjustment of thermophysical properties of base fluid. Nanoparticles in biological systems give new avenues for medical treatments. Currently, nanoparticles find their application in supplying different substances such as light, drugs, heat etc., to the specific types of cells, e.g., cancer cells. As an example, iron-based nanoparticles are useful as delivery vehicles for drug or radiation. Additionally, these iron particles are not harmful for nearby tissues [2]. Some biological applications of nanoparticles include proteins detection, pathogens biodetection, supply of drug and genes, fluorescent biological labels, and probing DNA structure etc. [3,4,5,6,7].

The above biomedical applications of nanofluids reveal that nanofluids can be transported by natural physiological mechanism such as peristaltic pumping. Peristaltic pumping means fluid transport via wave propagations along the length of flexible tube. This mechanism can be seen in chyme movement into the entrails, transport of semen in the tubes of male generative strip, pee transportation from kidney to the bladder, passage of the egg cell in the female fallopian tube, etc. Many peristaltic transport models have been developed in the past few decades where some basic novel analytical models for steady peristaltic flow [8] and unsteady peristaltic flow [9] have been investigated with Newtonian fluids under large wavelength and low Reynolds number approximations. A lot of earlier research studies on peristaltic motion have considered Newtonian fluids; however, non-Newtonian fluids [10,11,12,13,14,15] are also focused on more recently liquids where a nonlinear relation exists between shear rate and shear stress. Therefore, at a given pressure and temperature, the stickiness of a non-Newtonian liquid is not persistent but depends on the kinematics record of the liquid or on the shear rate [16]. Hence, a single fundamental relation cannot be developed that can forecast the occurrence of all non-Newtonian fluid behavior. The Johnson–Segalman (JS) fluid model was developed as an attempt to predict non-Newtonian effects. Later this model was used by [17] for peristaltic mechanisms and then it was modified for magnetohydrodynamic fluids in [18]. The JS fluid model is a viscoelastic model that allows non-affine distortions [19]. Furthermore, a nanofluids flow model [20] with peristaltic pumping has been investigated and the application of this model for drug-delivery systems and in the treatment of cancer as a tool of destroying unwanted tissues has been discussed. Most recently, some mathematical models [21,22,23,24,25] on peristaltic pumping along with nanofluids have been developed and parametric effects have been analyzed.

In nanofluid dynamics, some mathematical models [26,27,28,29,30] have been studied with various physical applications and various flow regimes. However, double diffusion (DD) is a convective phenomenon induced by two gradients with different (solutal and nanoparticle) densities and diffusion rates has not been considered in [26,27,28,29,30]. Considering the wide applications of double-diffusive convection, it has been examined in nanofluid flow models and with peristaltic pumping [31,32,33,34,35,36,37,38,39,40].

Based on the reviewed literature, it is identified that no single research has confirmed the partial slip impact of peristaltic flow of JS nanofluids with double-diffusive convection in an asymmetric channel by considering induced magnetic field. Therefore, the proposed research work is presented to fill this gap.

The paper is organized in different sections with the following details: Section 2 defines basic governing equations; Section 3 presents mathematical interpretations of JS fluid model for two-directional and two-dimensional movements of nanofluids under DD convection; Section 4 highlights the proposed work and demonstrates the graphical interpretation of acquired results; and Section 5 discusses the concluding remarks for the present problem.

## 2. Basic Equations

For a hydro-magnetic Johnson—Segalman nanofluid the basic equations are as: (a) Maxwell’s equation

(1) $$\nabla \cdot \tilde{E}=0,\;\nabla \cdot \tilde{H}=0,$$

(2) $$\nabla \times \tilde{H}=\tilde{J},\;\tilde{J}=\sigma \left\{\tilde{E}+\mu_{e}\left(V\times \tilde{H}\right)\right\},$$

(3) $$\nabla \times \tilde{E}=-\mu_{e}\frac{\partial \tilde{H}}{\partial t},$$

Now, the induction equation can be computed using Equations (1)–(3) as follows:

(4) $$\frac{\partial {\tilde{H}}^{+}}{\partial t}=\nabla \times \left(V\times {\tilde{H}}^{+}\right)+\frac{1}{\chi }\nabla^{2}{\tilde{H}}^{+}.$$

where $\mu_{e}$ symbolizes magnetic permeability, g indicates acceleration, σ stands for electric conductivity, t  indicates time, E is induced electric field, J  denotes current density, and V  stands for velocity vector. (b) Continuity equation is

(5) div V=0.

(c) Naiver–Stoke equation is

(6) $$\rho_{f}\left(\frac{dV}{dt}\right)=div\tau -\mu_{e}\left({\tilde{H}}^{+}\cdot \nabla \right){\tilde{H}}^{+}-\nabla \left(\frac{1}{2}\mu_{e}{\left({\tilde{H}}^{+}\right)}^{2}\right)+F_{g}\;\;$$

where $F_{g}$ is body force and is defined as

(7) $$F_{g}=g\{\left(1-\Theta_{0}\right)\rho_{f0}\left\{\beta_{T}\left(T-T_{0}\right)+\beta_{C}\left(C-C_{0}\right)\right\}-\left(\rho_{p}-\rho_{f0}\right)\left(\Theta -\Theta_{0}\right)\}.\;$$

The stress tensor for Johnson–Segalman fluid is defined by [17,18]

(8) τ=−PI+ϖ, 

(9) $$\varpi =2\tilde{\mu }W+S,$$

(10) $$S+\beta \left[\frac{dS}{dt}+S\left(W_{1}-\alpha W\right)+{\left(W_{1}-\alpha W\right)}^{T}S\right]=2\tilde{\eta }W,$$

(11) $$W=\frac{1}{2}\left(grad(V\right)+grad{\left(V\right)}^{*}),$$

(12) $$W_{1}=\frac{1}{2}\left(grad(V\right)-grad{\left(V\right)}^{*}),\;$$

where $\beta_{T}$ stands for volumetric thermal expansion coefficient, $\beta_{C}$ indicates volumetric solutal expansion coefficient, C is concentration, $\rho_{p}$ denotes nanoparticle mass density, $\rho_{f_{0}}$ represents density of fluid $T_{0}$, $\rho_{f}\;$ indicates density of fluid, Θ is volume fraction nanoparticle, T stands for temperature, $\left(\frac{d}{dt}\right)$ represents material derivative, I stands for identity tensor, β is relaxation time,$\;(\tilde{\mu },$ $\tilde{\eta })$ represents dynamic viscosities, α  is slip parameter, P indicates  pressure, and (W, $W_{1})$ represents velocity gradient symmetric and skew symmetric part.

Note that for α=1 and $\tilde{\mu }=0,$ model (10) corresponds to the Maxwell fluid model, whereas for $\tilde{\mu }=0=\beta$, we have the standard Navier–Stokes fluid model.

(d) Using the estimation of Oberbeck–Boussinesq, the thermal energy, solute concentration, and nanoparticle fraction [31] are analyzed as:

(13) $${\left(\rho c\right)}_{f}\left(\frac{dT}{dt}\right)=k\nabla^{2}T+{\left(\rho c\right)}_{p}\{D_{B}\left(\nabla \Theta \cdot \nabla T\right)+\left(\frac{D_{T}}{T_{0}}\right)\nabla T\cdot \nabla T\}+D_{TC}\nabla^{2}C,$$

(14) $$\frac{dC}{dt}=D_{S}\nabla^{2}C+D_{Ct}\nabla^{2}T,$$

(15) $$\frac{d\Theta }{dt}=D_{B}\nabla^{2}\Theta +\left(\frac{D_{T}}{T_{0}}\right)\nabla^{2}T,\;$$

where $D_{B}$ indicates Brownian diffusion coefficient, $D_{TC}$ symbolizes Dufour diffusively, $D_{T}$ denotes thermophoretic diffusion coefficient, $D_{CT}$ stands for Soret diffusively, $D_{s}$ is solutal diffusively, ${\left(\rho c\right)}_{p},$ represents heat capacity of fluid, k stands for thermal conductivity, and ${\left(\rho c\right)}_{f}$ is effective nanoparticle heat capacity.

## 3. Mathematical Formulation

Consider an electrically conducting incompressible Johnson–Segalman nanofluid in a two-dimensional infinite conduit with a non-uniform thickness and a sinusoidal wave moving along its wall. The wave motion is maintained along the *X*-axis, whereas the *Y*-axis is perpendicular to it. The induced magnetic field $H\left(h_{X}\left(X,Y,t\right),H_{0}+h_{Y}\left(X,Y,t\right),0\right)$ and the total magnetic field $H^{+}\left(h_{X}\left(X,Y,t\right),H_{0}+h_{Y}\left(X,Y,t\right),0\right)$ are produced by a sustained magnetic field of $H_{0}$ intensity acting perpendicularly. The problem’s geometry can be expressed mathematically as

(16) $$M\left(X,t\right)=\hat{b}\left(X\right)+\hat{a}\;sin\left(\frac{2\pi }{\lambda }\left(X-ct\right)\right),$$

where $\hat{b}\left(X\right)={\hat{b}}_{0}+{\hat{b}}_{1}X$ and $\left({\hat{b}}_{1}<<1\right)$ is constant. Moreover $\hat{b}$ symbolizes half-width of conduit at axial distance, λ is wavelength,$\;\hat{a}$ symbolizes wave amplitude, ${\hat{b}}_{0}$ inlet half-width, t denotes time, and c stands for wave speed.

Consider two-dimensional and directional flow velocity as:

(17) V=(U(X,Y,t),V(X,Y,t),0), 

Using Equation (17), the Equations (4)–(15) in laboratory frame (X,Y) become

(18) $$\frac{\partial U}{\partial X}+\frac{\partial V}{\partial Y}=0,\;$$

(19) $$\rho \left(\frac{\partial }{\partial t}+U\frac{\partial }{\partial X}+V\frac{\partial }{\partial Y}\right)U=-\frac{\partial P}{\partial X}+\tilde{\mu }\left(\frac{\partial^{2}U}{\partial X^{2}}+\frac{\partial^{2}U}{\partial Y^{2}}\right)+\frac{\partial S_{XX}}{\partial X}+\frac{\partial S_{XY}}{\partial Y}-\frac{\mu_{e}}{2}\left(\frac{\partial H^{{+}^{2}}}{\partial Y}\right)\;+\mu_{e}\left(h_{X}\frac{\partial h_{X}}{\partial X}+h_{Y}\frac{\partial h_{X}}{\partial Y}+H_{0}\frac{\partial h_{X}}{\partial Y}\right)+g\{\left(1-\Theta_{0}\right)\rho_{f0}\beta_{T}\left(T-T_{0}\right)+\beta_{C}\left(C-C_{0}\right)\;-\left(\rho_{p}-\rho_{f0}\right)\left(\Theta -\Theta_{0}\right)\},\;\;$$

(20) $$\rho \left(\frac{\partial }{\partial T}+U\frac{\partial }{\partial X}+V\frac{\partial }{\partial Y}\right)V=-\frac{\partial P}{\partial Y}+\tilde{\mu }\left(\frac{\partial^{2}V}{\partial X^{2}}+\frac{\partial^{2}V}{\partial Y^{2}}\right)+\frac{\partial S_{YX}}{\partial X}+\frac{\partial S_{YY}}{\partial Y}\;-\frac{\mu_{e}}{2}\left(\frac{\partial H^{{+}^{2}}}{\partial Y}\right)+\mu_{e}\left(h_{X}\frac{\partial h_{Y}}{\partial X}+h_{Y}\frac{\partial h_{Y}}{\partial Y}+H_{0}\frac{\partial h_{Y}}{\partial Y}\right),$$

(21) $${\left(\rho c\right)}_{f}\left(\frac{\partial }{\partial t}+U\frac{\partial }{\partial X}+V\frac{\partial }{\partial Y}\right)T=k\left(\frac{\partial^{2}T}{\partial X^{2}}+\frac{\partial^{2}T}{\partial Y^{2}}\right)+{\left(\rho c\right)}_{p}\{D_{B}\left(\frac{\partial \Theta }{\partial X}\frac{\partial T}{\partial X}+\frac{\partial \Theta }{\partial Y}\frac{\partial T}{\partial Y}\right)\;\left(\frac{D_{T}}{T_{0}}\right)\left[{\left(\frac{\partial T}{\partial X}\right)}^{2}+{\left(\frac{\partial T}{\partial Y}\right)}^{2}\right]\}+D_{TC}\left(\frac{\partial^{2}C}{\partial X^{2}}+\frac{\partial^{2}C}{\partial Y^{2}}\right),\;$$

(22) $$\left(\frac{\partial }{\partial t}+U\frac{\partial }{\partial X}+V\frac{\partial }{\partial Y}\right)C=D_{S}\left(\frac{\partial^{2}C}{\partial X^{2}}+\frac{\partial^{2}C}{\partial Y^{2}}\right)+D_{TC}\left(\frac{\partial^{2}T}{\partial X^{2}}+\frac{\partial^{2}T}{\partial Y^{2}}\right),\;$$

(23) $$\left(\frac{\partial }{\partial t}+U\frac{\partial }{\partial X}+V\frac{\partial }{\partial Y}\right)\Theta =D_{B}\left(\frac{\partial^{2}\Theta }{\partial X^{2}}+\frac{\partial^{2}\Theta }{\partial Y^{2}}\right)+\left(\frac{D_{T}}{T_{0}}\right)\left(\frac{\partial^{2}T}{\partial X^{2}}+\frac{\partial^{2}T}{\partial Y^{2}}\right),$$

The stress in component forms is defined as

(24) $$2\eta \frac{\partial U}{\partial X}=S_{XX}+\xi \left(\frac{\partial }{\partial t}+U\frac{\partial }{\partial X}+V\frac{\partial }{\partial Y}\right)S_{XX}-2\xi \beta S_{XX}\frac{\partial U}{\partial X}\;+\xi \left[\left(1-\beta \right)\frac{\partial V}{\partial X}-\left(1+\beta \right)\frac{\partial U}{\partial Y}\right]S_{XY},\;$$

(25) $$\eta \left(\frac{\partial U}{\partial Y}+\frac{\partial V}{\partial X}\right)=S_{XY}+\xi \left(\frac{\partial }{\partial t}+U\frac{\partial }{\partial X}+V\frac{\partial }{\partial Y}\right)S_{XY}+\frac{\xi }{2}\left[\left(1-\beta \right)\frac{\partial U}{\partial Y}-\left(1+\beta \right)\frac{\partial V}{\partial X}\right]S_{XX}\;+\frac{\xi }{2}\left[\left(1-\beta \right)\frac{\partial V}{\partial X}-\left(1+\beta \right)\frac{\partial U}{\partial Y}\right]S_{YY},$$

(26) $$2\eta \frac{\partial V}{\partial Y}=S_{YY}+\xi \left(\frac{\partial }{\partial t}+U\frac{\partial }{\partial X}+V\frac{\partial }{\partial Y}\right)S_{YY}-2\xi \beta S_{YY}\frac{\partial V}{\partial Y}\;+\xi \left[\left(1-\beta \right)\frac{\partial U}{\partial Y}-\left(1+\beta \right)\frac{\partial V}{\partial X}\right]S_{XY},\;$$

Now using Galilean transformations in fixed frame (X,Y) and wave frame (x,y) as

(27) v=V, u=U−c, y=Y, x=X−ct, p(x,y)=P(X,Y,t), 

and defining non-dimensional parameters

(28) $$\overset{¯}{y}=\frac{y}{{\hat{b}}_{0}},\;\overset{¯}{x}=\frac{x}{\lambda },\;\overset{¯}{v}=\frac{v}{c},\overset{¯}{u}=\frac{u}{c},\;\delta =\frac{{\hat{b}}_{0}}{\lambda },\;\overset{¯}{t}=\frac{ct}{\lambda },\;Re=\frac{\rho_{f}c{\tilde{b}}_{0}}{\tilde{\mu }},\;\overset{¯}{m}=\frac{M}{{\hat{b}}_{0}},\;Wi=\frac{c\beta }{{\hat{b}}_{0}},\;\theta =\frac{T-T_{0}}{T_{1}-T_{0}},\;\gamma =\frac{C-C_{0}}{C_{1}-C_{0}},\;\Omega =\frac{\Theta -\Theta_{0}}{\Theta_{1}-\Theta_{0}},\;u=\frac{\partial \Psi }{\partial y},\;v=-\delta \frac{\partial \Psi }{\partial x},\;h_{x}=\frac{\partial \Phi }{\partial y},\;h_{y}=-\delta \frac{\partial \Phi }{\partial x},\;Pr=\frac{{\left(\rho c\right)}_{f}\;\upsilon }{k},\;Ln=\frac{\upsilon }{D_{B}},\;N_{CT}=\frac{D_{CT}\left(T_{1}-T_{0}\right)}{\left(C_{1}-C_{0}\right)D_{S}},\;N_{TC}=\frac{D_{CT}\left(C_{1}-C_{0}\right)}{k\left(T_{1}-T_{0}\right)},\overset{¯}{p}=\frac{{\hat{b}}_{0}^{2}p\left(x\right)}{\left(\tilde{\mu }+\tilde{\eta }\right)c\lambda },\;G_{rt}=\frac{g{\hat{b}}_{0}^{2}\left(1-\Theta_{0}\right)\left(T_{1}-T_{0}\right)\rho_{f0}\beta_{T}}{\tilde{\mu }c},\;G_{rc}=\frac{g\left(1-\Theta_{0}\right)\rho_{f0}\beta_{c}\left(C_{1}-C_{0}\right){\hat{b}}_{0}^{2}}{\tilde{\mu }c},\;\overset{¯}{S}=\frac{{\hat{b}}_{0}}{\tilde{\mu }c}S,\;Le=\frac{\upsilon }{D_{S}},\;N_{b}=\frac{{\left(\rho c\right)}_{p}D_{B}\left(\Theta_{1}-\Theta_{0}\right)}{k},\;N_{t}=\frac{{\left(\rho c\right)}_{p}D_{T}\left(T_{1}-T_{0}\right)}{T_{0}k},G_{rF}=\frac{g\left(\rho_{p}-\rho_{f0}\right)\left(\Theta_{1}-\Theta_{0}\right)}{\tilde{\mu }c}{\hat{b}}_{0}^{2},\;$$

where $G_{rF}$ stands for Grashof number of nanoparticle, $G_{rt}$ represents thermal Grashof number, $G_{rc}$ denotes solutal Grashof number, θ stands for temperature, Ω is nanoparticle fraction, γ stands for solutal (species) concentration, $N_{b}$ symbolizes Brownian motion, Le denotes Lewis number, $N_{t}$ indicates thermophoresis parameter, Pr represents Prandtl number, Ln indicates nanofluid Lewis number, $N_{TC}$ denotes parameter of Dufour, $N_{CT}$ stands for Soret parameter, δ indicates wave number, and Re represents Reynolds number.

The Equation (16) in dimensionless form becomes

(29) m(x)=1+ξx+ε sin(2πx),

where $\varepsilon =\frac{\hat{b}}{{\hat{b}}_{0}}$ indicates occlusion or amplitude ratio and $\xi =\frac{{\hat{b}}_{1}}{{\hat{b}}_{0}}.$

Equations (27) and (28) automatically satisfy Equation (18), and Equations (19)–(26) in the wave frame (by dropping bars) become

(30) $$Re\;\delta \left(\psi_{xy}\psi_{y}-\psi_{yy}\psi_{x}\right)=-\left(\frac{\tilde{\mu }+\tilde{\eta }}{\tilde{\mu }}\right)\frac{\partial p}{\partial x}+\delta \frac{\partial S_{xx}}{\partial x}+\frac{\partial S_{xy}}{\partial y}+\left(\delta^{2}\frac{\partial^{3}\psi }{\partial x^{2}\partial y}+\frac{\partial^{3}\psi }{\partial y^{3}}\right),\;+Re\;S_{1}^{2}\Phi_{yy}+ReS_{1}^{2}\delta \left(\Phi_{y}\Phi_{xy}-\Phi_{x}\Phi_{yy}\right)+G_{rT}\theta +G_{rc}\gamma -G_{rF}\Omega ,$$

(31) $$Re\;\delta^{3}\left(\psi_{xy}\psi_{x}-\psi_{xx}\psi_{y}\right)=-\left(\frac{\tilde{\mu }+\tilde{\eta }}{\tilde{\mu }}\right)\frac{\partial p}{\partial y}+\delta^{2}\frac{\partial S_{xy}}{\partial x}+\delta \frac{\partial S_{yy}}{\partial y}-\delta^{2}\left(\delta^{2}\frac{\partial^{3}\psi }{\partial x^{3}}+\frac{\partial^{3}\psi }{\partial x\partial y^{2}}\right)\;-Re\;\delta^{2}S_{1}^{2}\Phi_{xy}-ReS_{1}^{2}\delta^{3}\left(\Phi_{y}\Phi_{xx}-\Phi_{x}\Phi_{xy}\right),\;$$

(32) $$Re\;Pr\;\delta \left(\theta_{x}\psi_{y}-\theta_{y}\psi_{x}\right)=\left(\theta_{yy}+\delta^{2}\theta_{xx}\right)+N_{TC}\left(\delta^{2}\gamma_{xx}+\gamma_{yy}\right)\;+N_{b}\left(\delta^{2}\Omega_{x}\theta_{x}+\theta_{y}\Omega_{y}\right)+N_{t}\left(\delta^{2}{\left(\theta_{x}\right)}^{2}+{\left(\theta_{y}\right)}^{2}\right),$$

(33) $$Re\;\delta \;Le\left(\gamma_{x}\psi_{y}-\gamma_{y}\psi_{x}\right)=\left(\delta^{2}\gamma_{xx}+\gamma_{yy}\right)+N_{CT}\left(\delta^{2}\theta_{xx}+\theta_{yy}\right),\;\;\;$$

(34) $$Re\;\delta \;Ln\left(\psi_{y}\Omega_{x}-\psi_{x}\Omega_{y}\right)=\left(\delta^{2}\Omega_{xx}+\Omega_{yy}\right)+\frac{N_{t}}{N_{b}}\left(\delta^{2}\theta_{xx}+\theta_{yy}\right),\;\;$$

(35) $$\Psi_{y}-\delta \left(\Phi_{x}\Psi_{y}-\Phi_{y}\Psi_{x}\right)+\frac{1}{R_{m}}\left(\delta^{2}\Phi_{xx}+\Phi_{yy}\right)=E.$$

(36) $$\left(\frac{2\delta \tilde{\eta }}{\tilde{\mu }}\right)\frac{\partial^{2}\psi }{\partial x\partial y}=S_{xx}+\delta Wi\left(\frac{\partial \psi }{\partial y}\frac{\partial }{\partial x}-\frac{\partial \psi }{\partial x}\frac{\partial }{\partial y}\right)S_{xx}-2\alpha Wi\delta \frac{\partial^{2}\psi }{\partial x\partial y}\;-Wi\left(\delta^{2}\left(1-\alpha \right)\frac{\partial^{2}\psi }{\partial x^{2}}+\left(1+\alpha \right)\frac{\partial^{2}\psi }{\partial y^{2}}\right)S_{xy},$$

(37) $$\frac{\tilde{\eta }}{\tilde{\mu }}\left(\frac{\partial^{2}\psi }{\partial y^{2}}-\delta^{2}\frac{\partial^{2}\psi }{\partial x^{2}}\right)=S_{xy}+Wi\delta \left(\frac{\partial \psi }{\partial y}\frac{\partial }{\partial x}-\frac{\partial \psi }{\partial x}\frac{\partial }{\partial y}\right)S_{xy}\;+\frac{Wi}{2}\left(\left(1-\alpha \right)\frac{\partial^{2}\psi }{\partial y^{2}}+\delta^{2}\left(1+\alpha \right)\frac{\partial^{2}\psi }{\partial x^{2}}\right)S_{xx}-\frac{Wi}{2}\left(\delta^{2}\left(1-\alpha \right)\frac{\partial^{2}\psi }{\partial x^{2}}+\left(1+\alpha \right)\frac{\partial^{2}\psi }{\partial y^{2}}\right)S_{yy},\;$$

(38) $$-\left(\frac{2\delta \tilde{\eta }}{\tilde{\mu }}\right)\frac{\partial^{2}\psi }{\partial x\partial y}=S_{yy}+Wi\delta \left(\frac{\partial \psi }{\partial y}\frac{\partial }{\partial x}-\frac{\partial \psi }{\partial x}\frac{\partial }{\partial y}\right)S_{yy}+2\alpha \delta Wi\frac{\partial^{2}\psi }{\partial x\partial y}S_{yy}\;+Wi\left(\left(1-\alpha \right)\frac{\partial^{2}\psi }{\partial y^{2}}+\delta^{2}\left(1+\alpha \right)\frac{\partial^{2}\psi }{\partial x^{2}}\right)S_{xy},\;$$

Now applying the lubricant approach (δ<<1) and low but finite Reynolds number the Equations (30)–(38) are now simplified as

(39) $$0=-\left(\frac{\tilde{\mu }+\tilde{\eta }}{\tilde{\mu }}\right)\frac{\partial p}{\partial x}+\frac{\partial S_{xy}}{\partial y}+\frac{\partial^{3}\psi }{\partial y^{3}}+Re\;S_{1}^{2}\Phi_{yy}+G_{rt}\theta +G_{rc}\gamma -G_{rF}\Omega ,\;$$

(40) $$0=-\frac{\partial p}{\partial y},\;$$

(41) $$\frac{\partial^{2}\theta }{\partial y^{2}}+N_{TC}\frac{\partial^{2}\gamma }{\partial y^{2}}+N_{b}\left(\frac{\partial \Omega }{\partial y}\frac{\partial \theta }{\partial y}\right)+N_{t}{\left(\frac{\partial \theta }{\partial y}\right)}^{2}=0,$$

(42) $$\frac{\partial^{2}\gamma }{\partial y^{2}}+N_{CT}\frac{\partial^{2}\theta }{\partial y^{2}}=0,\;$$

(43) $$\frac{\partial^{2}\Omega }{\partial y^{2}}+\frac{N_{t}}{N_{b}}\frac{\partial^{2}\theta }{\partial y^{2}}=0,$$

(44) $$\frac{\partial^{2}\Phi }{\partial y^{2}}=R_{m}\left(E-\frac{\partial \Psi }{\partial y}\right).$$

where

(45) $$S_{xx}=Wi\left(1+\alpha \right)\frac{\partial^{2}\psi }{\partial y^{2}}S_{xy},\;\;$$

(46) $$\left(\frac{\tilde{\eta }}{\tilde{\mu }}\right)\frac{\partial^{2}\psi }{\partial y^{2}}=S_{xy}+\frac{Wi}{2}\left(1-\alpha \right)\frac{\partial^{2}\psi }{\partial y^{2}}S_{xx}-\frac{Wi}{2}\left(1+\alpha \right)\frac{\partial^{2}\psi }{\partial y^{2}}S_{yy},\;\;$$

(47) $$S_{yy}=-Wi\left(1-\alpha \right)\frac{\partial^{2}\psi }{\partial y^{2}}S_{xy},$$

Now eliminating pressure from Equations (39) and (40) and employing Equations (45)–(47), we get the following expression as:

(48) $$\frac{\partial^{2}S_{xy}}{\partial y^{2}}+\frac{\partial^{4}\psi }{\partial y^{4}}+Re\;S_{1}^{2}\Phi_{yyy}+G_{rt}\frac{\partial \theta }{\partial y}+G_{rc}\frac{\partial \gamma }{\partial y}-G_{rF}\frac{\partial \Omega }{\partial y}=0,$$

(49) $$S_{xy}=\frac{\left(\frac{\tilde{\eta }}{\tilde{\mu }}\right)\left(\frac{\partial^{2}\psi }{\partial y^{2}}\right)}{1+Wi^{2}\left(1-\alpha^{2}\right){\left(\frac{\partial^{2}\psi }{\partial y^{2}}\right)}^{2}},\;$$

(50) $$\frac{\partial^{2}}{\partial y^{2}}\left[\frac{\left(\frac{\tilde{\eta }}{\tilde{\mu }}+1\right)\left(\frac{\partial^{2}\psi }{\partial y^{2}}\right)+Wi^{2}\left(1-\alpha^{2}\right){\left(\frac{\partial^{2}\psi }{\partial y^{2}}\right)}^{3}}{1+Wi^{2}\left(1-\alpha^{2}\right){\left(\frac{\partial^{2}\psi }{\partial y^{2}}\right)}^{2}}\right]+Re\;S_{1}^{2}\Phi_{yyy}+G_{rt}\frac{\partial \theta }{\partial y}\;+G_{rc}\frac{\partial \gamma }{\partial y}-G_{rF}\frac{\partial \Omega }{\partial y}=0,\;$$

(51) $$\left(\frac{\tilde{\mu }+\tilde{\eta }}{\tilde{\mu }}\right)\frac{\partial p}{\partial x}=\frac{\partial }{\partial y}\left[\frac{\left(\frac{\tilde{\eta }}{\tilde{\mu }}\right)\left(\frac{\partial^{2}\psi }{\partial y^{2}}\right)}{1+Wi^{2}\left(1-\alpha^{2}\right){\left(\frac{\partial^{2}\psi }{\partial y^{2}}\right)}^{2}}\right]+\frac{\partial^{3}\psi }{\partial y^{3}}\;+Re\;S_{1}^{2}\Phi_{yy}+G_{rt}\theta +G_{rc}\gamma -G_{rF}\Omega ,\;$$

Equations (50) and (51) can be simplified after employing binomial expansion for small $Wi^{2}$ as follows:

(52) $$\frac{\partial^{2}}{\partial y^{2}}\left[\frac{\partial^{2}\psi }{\partial y^{2}}+\lambda_{1}Wi^{2}{\left(\frac{\partial^{2}\psi }{\partial y^{2}}\right)}^{3}+\lambda_{2}Wi^{4}{\left(\frac{\partial^{2}\psi }{\partial y^{2}}\right)}^{5}\right]+Re\;S_{1}^{2}\Phi_{yyy}\;+G_{rt}\frac{\partial \theta }{\partial y}+G_{rc}\frac{\partial \gamma }{\partial y}-G_{rF}\frac{\partial \Omega }{\partial y}=0$$

(53) $$\frac{\partial^{2}}{\partial y^{2}}\left[\frac{\partial^{2}\psi }{\partial y^{2}}+\lambda_{1}Wi^{2}{\left(\frac{\partial^{2}\psi }{\partial y^{2}}\right)}^{3}+\lambda_{2}Wi^{4}{\left(\frac{\partial^{2}\psi }{\partial y^{2}}\right)}^{5}\right]-Re\;S_{1}^{2}R_{m}\frac{\partial^{2}\Psi }{\partial y^{2}}\;+G_{rt}\frac{\partial \theta }{\partial y}+G_{rc}\frac{\partial \gamma }{\partial y}-G_{rF}\frac{\partial \Omega }{\partial y}=0,\;\;$$

(54) $$\frac{\partial p}{\partial x}=\frac{\partial^{3}\psi }{\partial y^{3}}+\lambda_{1}Wi^{2}\frac{\partial }{\partial y}\left[{\left(\frac{\partial^{2}\psi }{\partial y^{2}}\right)}^{3}\right]+\lambda_{2}Wi^{4}\frac{\partial }{\partial y}\left[{\left(\frac{\partial^{2}\psi }{\partial y^{2}}\right)}^{5}\right]\;+Re\;S_{1}^{2}\Phi_{yy}+G_{rt}\theta +G_{rc}\gamma -G_{rF}\Omega ,\;$$

The boundary constrains for the stated problem in wave frame are defined as follows:

(55) $$\Psi =0,\;\frac{\partial^{2}\Psi }{\partial y^{2}}=0\;\mathrm{on}\;y=0,$$

(56) $$\Psi =F,\;\frac{\partial \Psi }{\partial y}=-1\;\mathrm{on}\;y=m\left(x\right)=1+\xi x+\varepsilon \;sin(2\pi x),\;$$

(57) $$\frac{\partial \Phi }{\partial y}=0\;\mathrm{on}\;y=0\;\mathrm{and}\;\Phi =0\;\mathrm{on}\;y=m\left(x\right),$$

(58) θ=0 on y=0 and θ=1 on y=m(x), 

(59) Ω=0 on y=0 and Ω=1 on y=m(x), 

(60) γ=0 on y=0 and γ=1 on y=m(x).    

Here, F is the time mean wave frame flow rate, which is connected to the time mean flow (Q) by the equations Q=F+1 and $F=\int_{0}^{m}\frac{\partial \Psi }{\partial y}dy.$

Special Cases

(a) In the non-existence of the induced magnetic field, the result of [31] can be recovered as a special case of the intended problem when $\xi =0,\;\lambda_{1}=0,\;\lambda_{2}=0$.
(b) In the absence of the induced magnetic field, the result of [8] can be found as a special case of the intended problem when $\xi =0,\;\lambda_{1}=0,\;\lambda_{2}=0,G_{rF}=0,\;G_{rt}=0,\;G_{rc}=0.$

## 4. Solution of Problem

### 4.1. Exact Solution

The exact solution of Equations (41)–(43) that meets the boundary constraints (58)–(60) is

(61) $$\theta =\frac{e^{-yl}-1}{e^{-hl}-1}.$$

(62) $$\Omega =\frac{N_{t}\left(e^{-yl}-1\right)}{N_{b}\left(1-e^{-hl}\right)}+\frac{\left(\frac{N_{t}}{N_{b}}+1\right)y}{h}.\;$$

(63) $$\gamma =\frac{N_{\mathrm{CT}}\left(e^{-yl}-1\right)}{1-e^{-hl}}+\frac{\left(N_{\mathrm{CT}}+1\right)y}{h}.\;$$

where

(64) $$l=\frac{N_{t}+N_{b}}{h\left(1-N_{\mathrm{CT}}N_{\mathrm{TC}}\right)}.$$

### 4.2. Numerical Solution

The nonlinear nature of partial differential equations (PDE’s) makes exact solutions of Equations (44), (53) and (54) challenging. The numerical results of the nonlinear PDEs are determined using software Mathematica. As a result of the numerical approximation of solutions, graphical visualization is performed to discuss the result’s validity.

**Figure 1:**
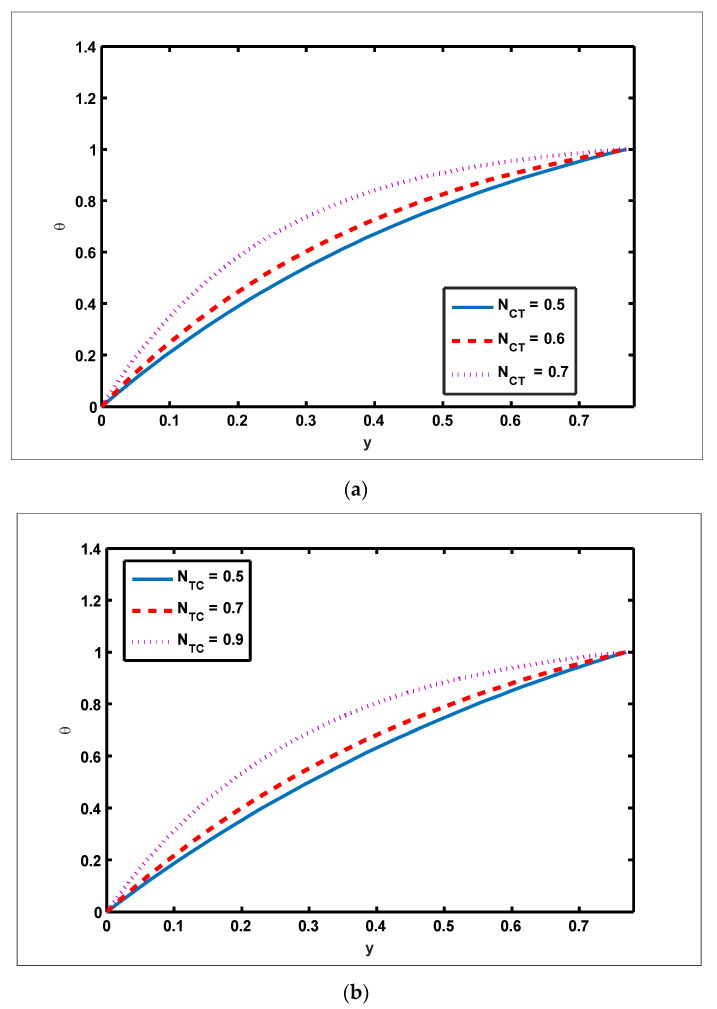

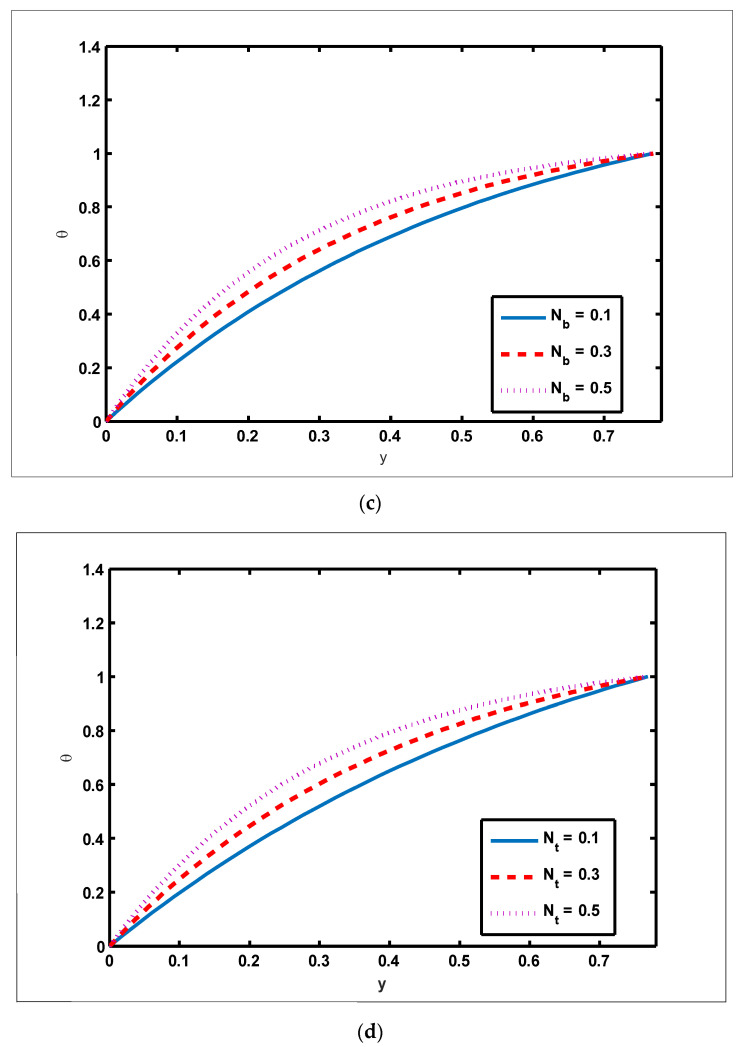
Temperature profile for Soret, Dufour, Brownian motion, and thermophoresis parameters. Values of other physical parameters are (**a**) $N_{t}=0.3,\;N_{TC}=1.2,\;N_{b}=0.3,\;b=0.7,\;a=0.3,\;x=0.6.$ (**b**) $N_{b}=0.2,\;N_{t}=0.3,\;N_{CT}=0.9,\;b=0.7,\;a=0.3,\;x=0.6.$ (**c**) $N_{t}=0.3,\;N_{TC}=1.2,\;N_{CT}=0.9,\;b=0.7,\;a=0.3,\;x=0.6.$ (**d**) $N_{b}=0.2,\;N_{TC}=1.2,\;N_{CT}=0.9,\;b=0.7,\;a=0.3,\;x=0.6$.

**Figure 2:**
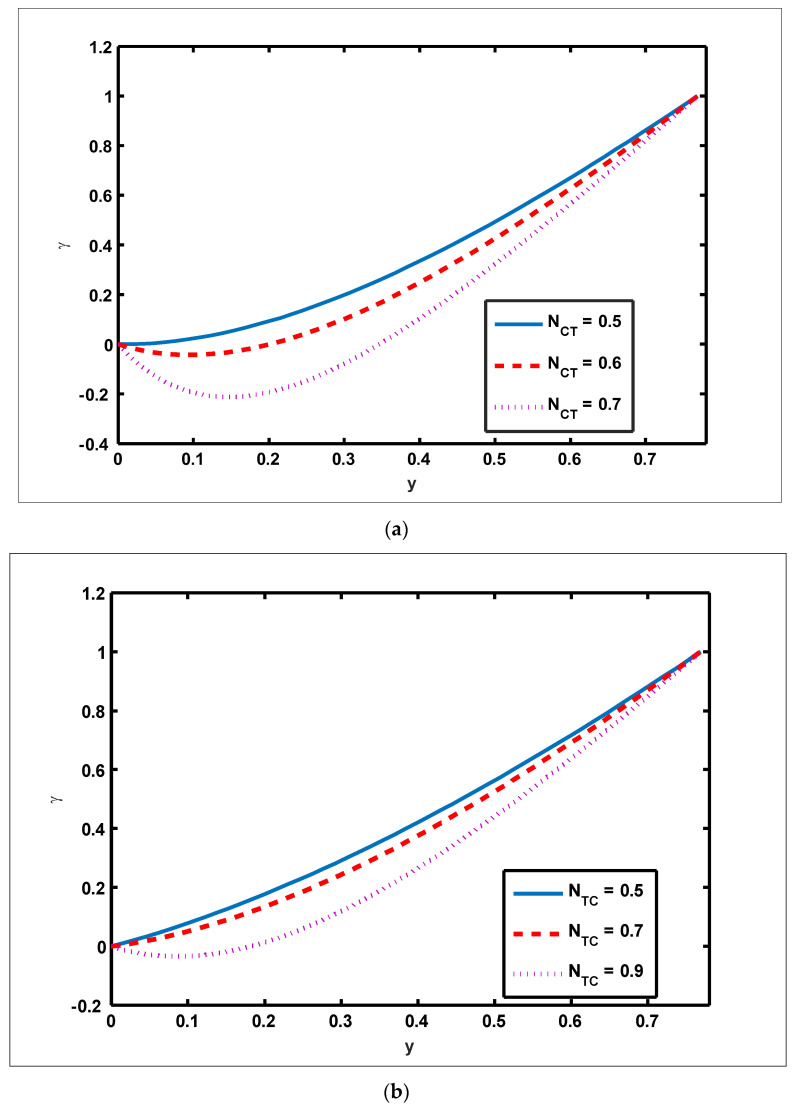

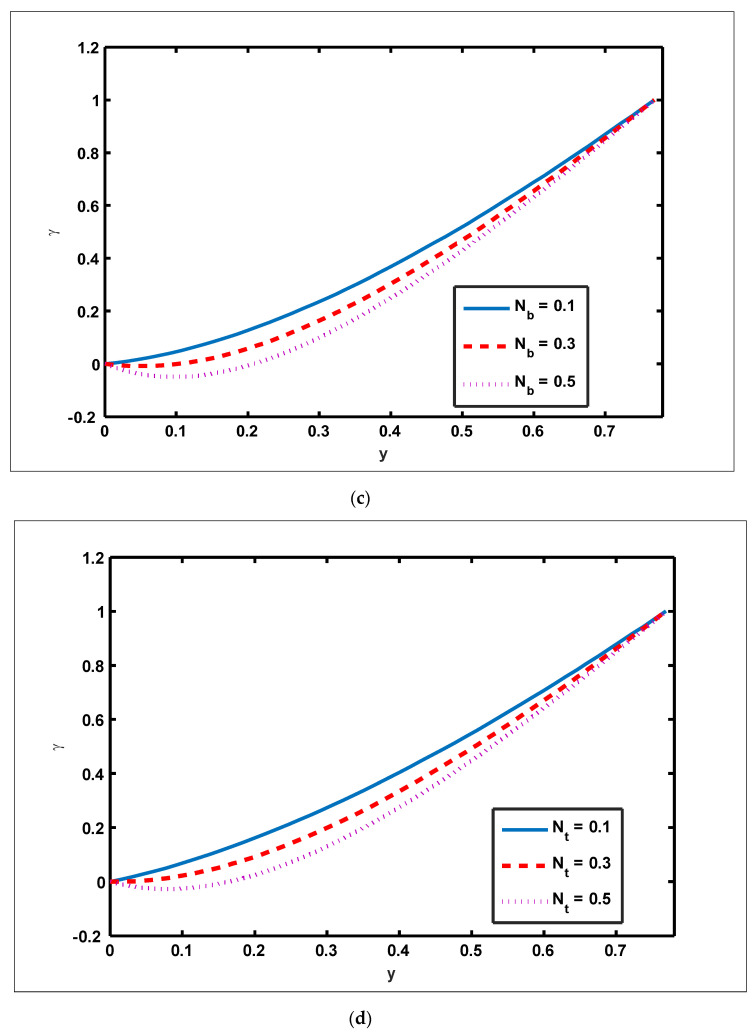
Concentration profile for Soret, Dufour, Brownian motion, and thermophoresis parameters. Values of other physical parameters are (**a**) $N_{t}=0.3,\;N_{TC}=1.2,\;N_{b}=0.3,\;b=0.7,\;a=0.3,\;x=0.6.$ (**b**) $N_{b}=0.2,\;N_{t}=0.3,\;N_{CT}=0.9,\;b=0.7,\;a=0.3,\;x=0.6.$ (**c**) $N_{t}=0.3,\;N_{TC}=1.2,\;N_{CT}=0.9,\;b=0.7,\;a=0.3,\;x=0.6.$ (**d**) $N_{b}=0.2,\;N_{TC}=1.2,\;N_{CT}=0.9,\;b=0.7,\;a=0.3,\;x=0.6$.

**Figure 3:**
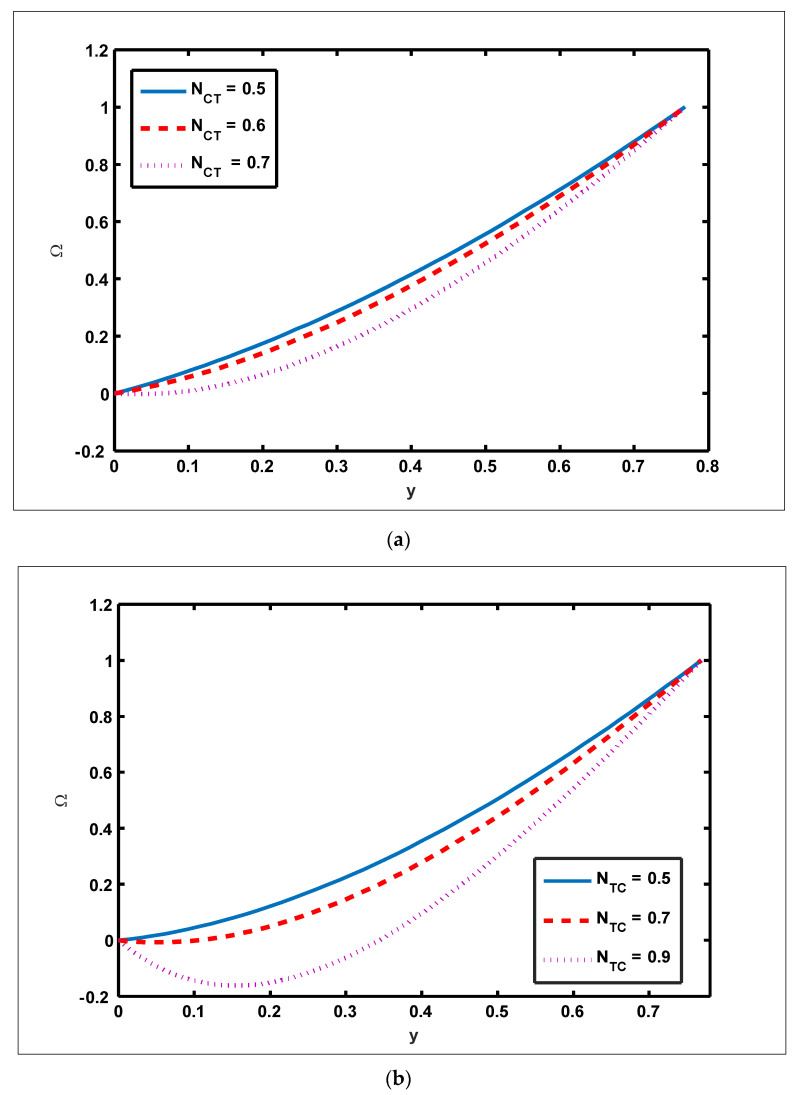

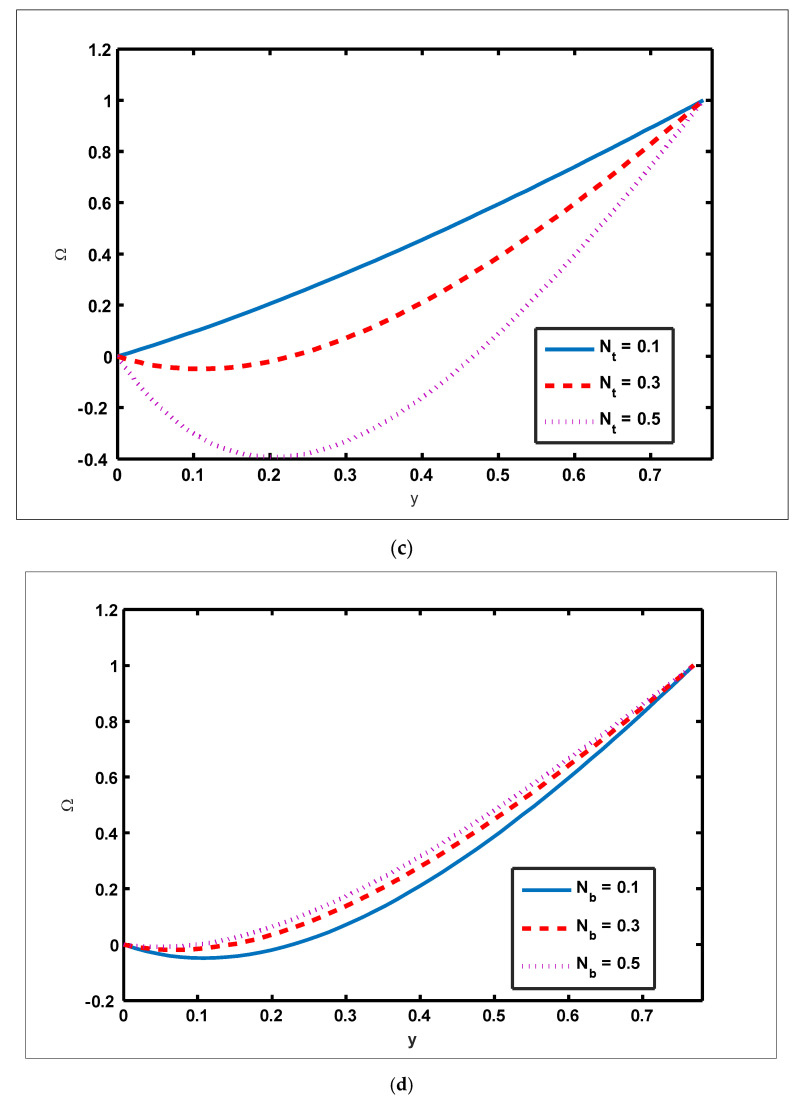
Profile of nanoparticle fraction for Soret, Dufour, thermophoresis, and Brownian motion parameters. Values of other physical parameters are (**a**) $N_{t}=0.3,\;N_{TC}=1.2,\;N_{b}=0.3,\;b=0.7,\;a=0.3,\;x=0.6.$ (**b**) $N_{b}=0.2,\;N_{t}=0.3,\;N_{CT}=0.9,\;b=0.7,\;a=0.3,\;x=0.6.$ (**c**) $N_{t}=0.3,\;N_{TC}=1.2,\;N_{CT}=0.9,\;b=0.7,\;a=0.3,\;x=0.6.$ (**d**) $N_{b}=0.2,\;N_{TC}=1.2,\;N_{CT}=0.9,\;b=0.7,\;a=0.3,\;x=0.6$.

**Figure 4:**
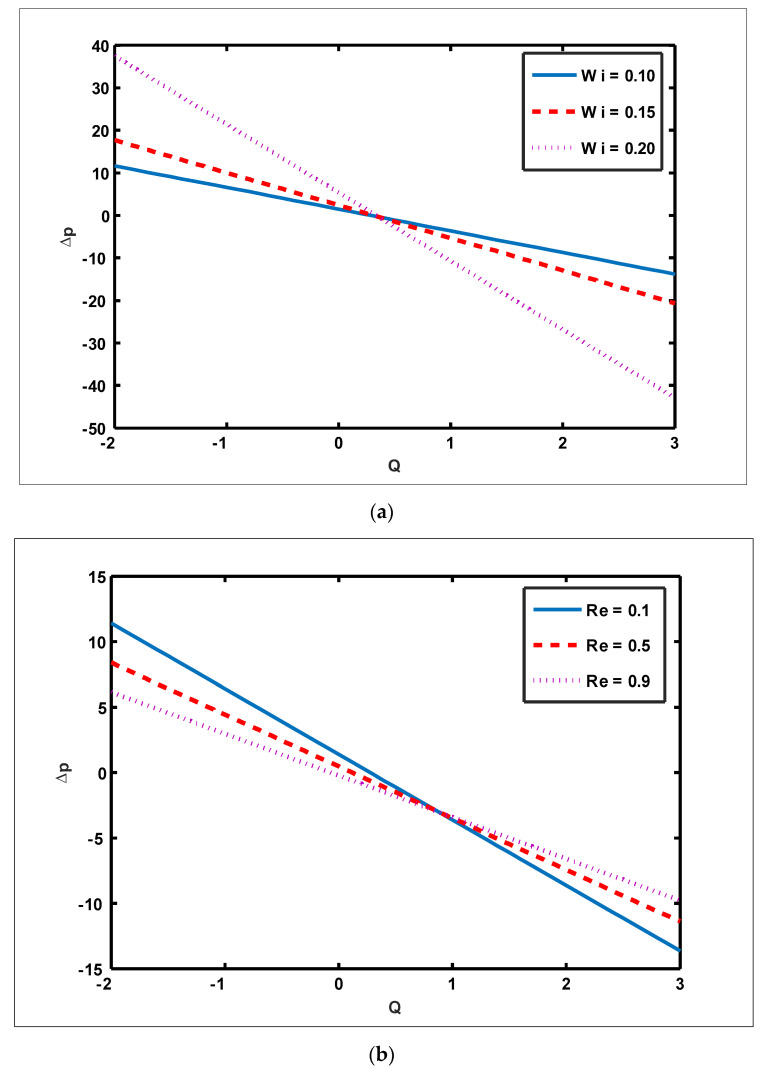

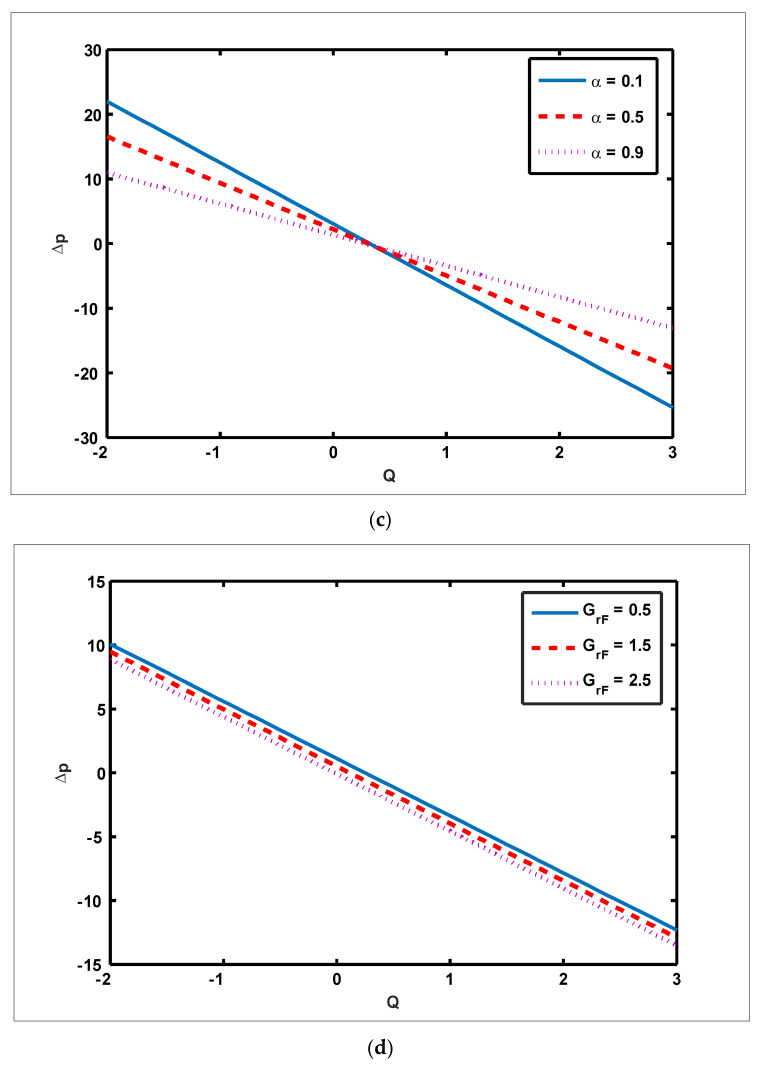
Effect of Weissenberg number, Reynolds number, slip parameter, and Grashof number of nanoparticles on pressure rise. (**a**) $N_{t}=0.3,\;N_{TC}=1.2,\;N_{b}=0.4,\;b=0.7,\;a=0.3,\;N_{CT}=0.9,\;x=0.1,\;Re=0.5,\;S_{1}=0.3,\;R_{m}=0.5,\;E=0.5,\;G_{rc}=1,\;G_{rt}=0.5,\;G_{rF}=0.8,\;\alpha =0.8,\tilde{\eta }=1,\;\tilde{\mu }=1\;$. (**b**) $N_{t}=0.3,\;N_{TC}=1.2,\;N_{b}=0.4,\;b=0.7,\;a=0.3,\;N_{CT}=0.9,\;x=0.1,\;S_{1}=0.3,\;R_{m}=0.5,\;E=0.5,\;G_{rc}=1,\;G_{rt}=0.5,\;G_{rF}=0.8,\;\alpha =0.8,\;\tilde{\eta }=1,\;\tilde{\mu }=1\;$. (**c**) $N_{t}=0.3,\;N_{TC}=1.2,\;N_{b}=0.4,\;b=0.7,\;Wi=0.3,\;N_{CT}=0.9,\;x=0.1,\;Re=0.5,\;S_{1}=0.3,\;R_{m}=0.5,\;E=0.5,\;G_{rc}=1,\;G_{rt}=0.5,\;G_{rF}=0.8,\;\alpha =0.8,\;\tilde{\eta }=1,\;\tilde{\mu }=1$ (**d**) $N_{t}=0.3,\;N_{TC}=1.2,\;N_{b}=0.4,\;b=0.7,\;a=0.3,\;N_{CT}=0.9,\;x=0.1,\;Re=0.5,\;S_{1}=0.3,\;R_{m}=0.5,\;E=0.5,\;G_{rc}=1,\;G_{rT}=0.5,\;Wi=0.1,\;\alpha =0.8,\;\tilde{\eta }=1,\;\tilde{\mu }=1$.

**Figure 5:**
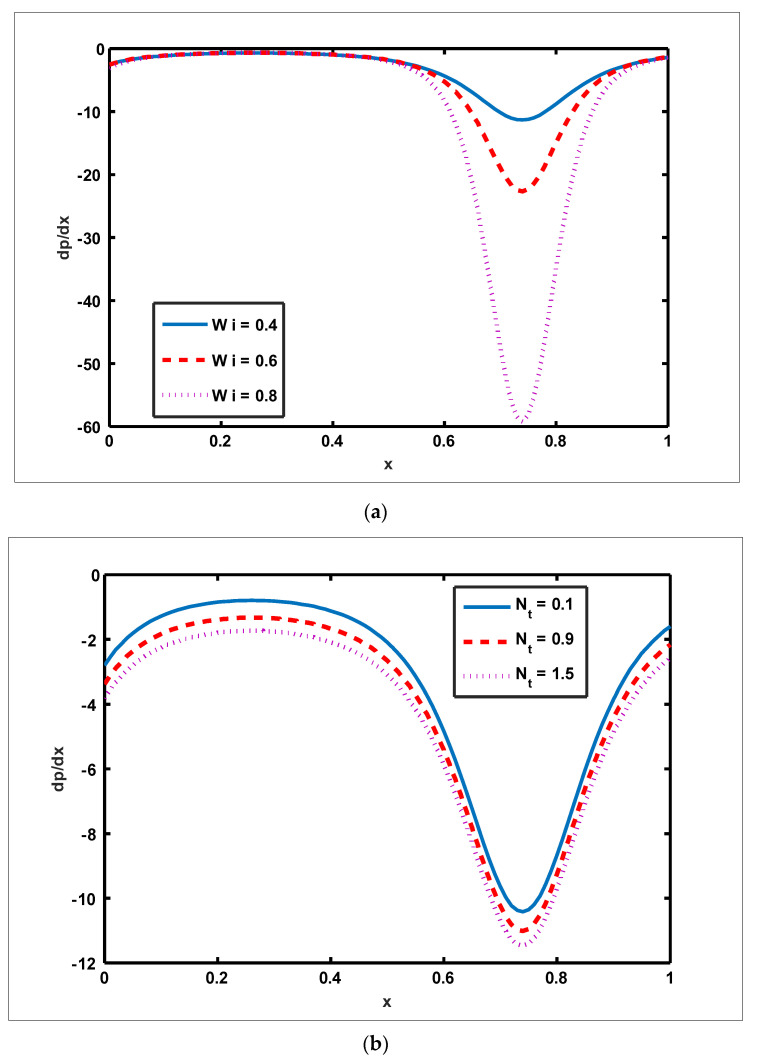

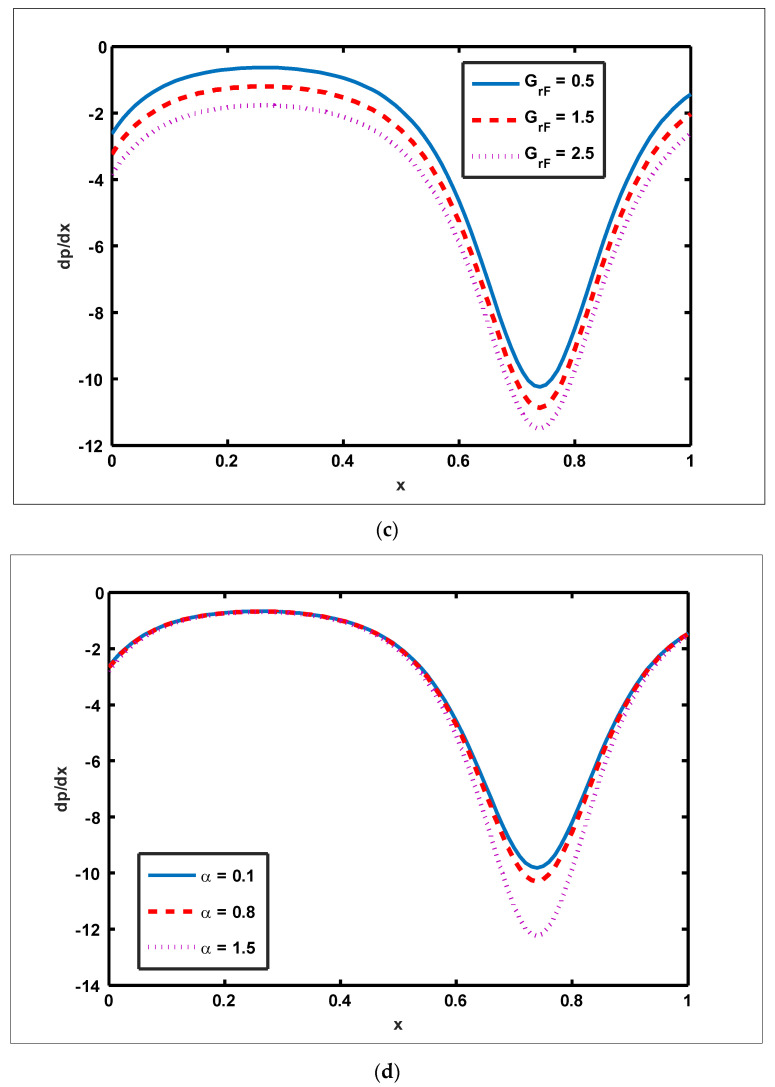
Effect of Weissenberg number, thermophoresis, Grashof number of nanoparticles, and slip parameter on pressure gradient. Values of other physical parameters are (**a**) $N_{t}=0.3,\;N_{TC}=1.2,\;N_{b}=0.4,\;b=0.7,\;a=0.3,\;N_{CT}=0.9,\;Q=1,\;Re=0.5,\;S_{1}=0.3,\;R_{m}=0.5,\;E=0.5,\;G_{rc}=1,\;G_{rt}=0.5,\;G_{rF}=0.8,\;\alpha =0.8,\;\tilde{\eta }=1,\;\tilde{\mu }=1$. (**b**) $Wi=0.1,\;N_{TC}=1.2,\;N_{b}=0.4,\;b=0.7,\;a=0.3,\;N_{CT}=0.9,\;Q=1,\;Re=0.5,\;S_{1}=0.3,\;R_{m}=0.5,\;E=0.5,\;G_{rc}=1,\;G_{rt}=0.5,\;Wi=0.1,\;\alpha =0.8,\;\tilde{\eta }=1,\;\tilde{\mu }=1$. (**c**) $N_{t}=0.3,\;N_{TC}=1.2,\;N_{b}=0.4,\;b=0.7,\;a=0.3,\;N_{CT}=0.9,\;Q=1,\;Re=0.5,\;S_{1}=0.3,\;R_{m}=0.5,\;E=0.5,\;G_{rc}=1,\;G_{rt}=0.5,\;G_{rF}=0.8,\;\alpha =0.8,\;\tilde{\eta }=1,\;\tilde{\mu }=1$. (**d**) $N_{t}=0.3,\;N_{TC}=1.2,\;N_{b}=0.4,\;b=0.7,\;a=0.3,\;N_{CT}=0.9,\;Q=1,\;Re=0.5,\;S_{1}=0.3,\;R_{m}=0.5,\;E=0.5,\;G_{rc}=1,\;G_{rt}=0.5,\;G_{rF}=0.8,\;Wi=0.1,\;\tilde{\eta }=1,\;\tilde{\mu }=1$.

**Figure 6:**
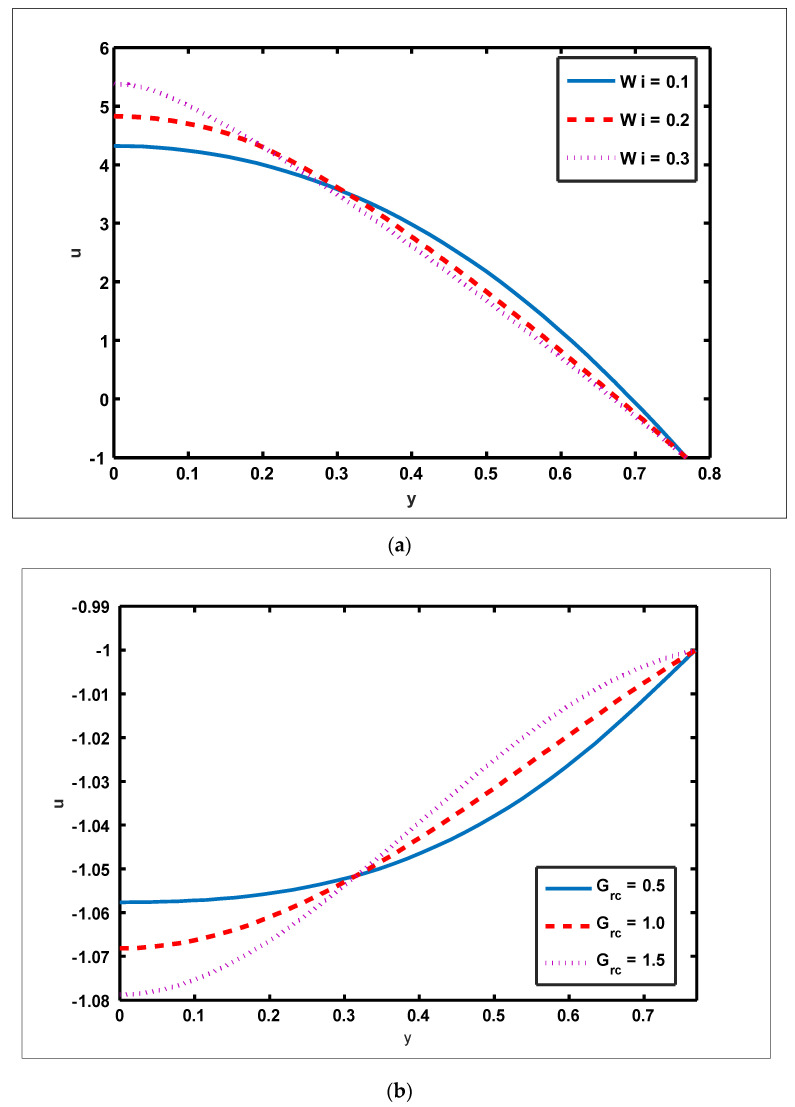

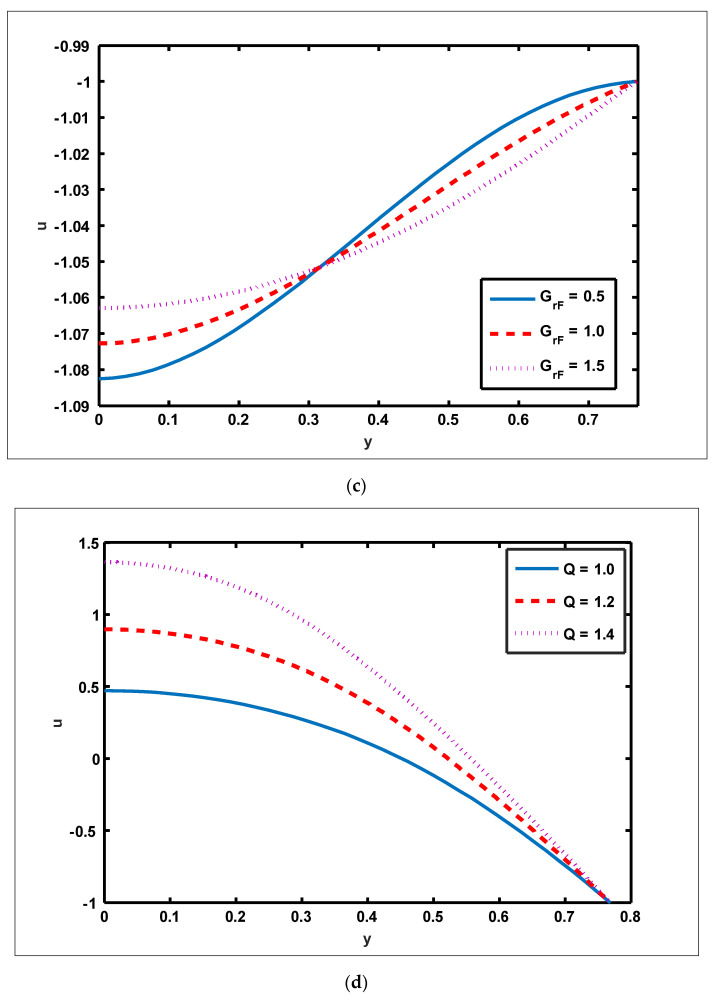
Velocity profile for Weissenberg number, solutal Grashof number, Grashof number of nanoparticles, and volume flow rate. Values of other physical parameters are (**a**) $N_{t}=0.3,\;N_{TC}=1.2,\;N_{b}=0.4,\;b=0.7,\;a=0.3,\;N_{CT}=0.9,\;Re=0.5,\;S_{1}=0.3,\;R_{m}=0.5,\;G_{rc}=1.4,\;x=0.6,\;G_{rt}=0.5,\;G_{rF}=0.8,$ $\alpha =0.8,\;Q=3,\;\tilde{\eta }=1,\;\tilde{\mu }=1\;$. (**b**) $N_{t}=0.3,\;N_{TC}=1.2,\;N_{b}=0.4,\;b=0.7,\;a=0.3,\;N_{CT}=0.9,\;Re=0.5,\;S_{1}=0.3,\;R_{m}=0.5,\;Wi=0.1,\;x=0.6,\;G_{rt}=0.5,\;G_{rF}=0.8,$ $\alpha =0.8,\;Q=3,\;\tilde{\eta }=1,\;\tilde{\mu }=1.\;\left(c\right)\;N_{t}=0.3,\;N_{TC}=1.2,\;N_{b}=0.4,\;b=0.7,\;a=0.3,\;N_{CT}=0.9,\;Re=0.5\;,S_{1}=0.3\;,R_{m}=0.5,\;G_{rc}=1.4,\;x=0.6,\;G_{rt}=0.5,\;Wi=0.1,\;\alpha =0.8,\;Q=3,\;\tilde{\eta }=1,\;\tilde{\mu }=1.\;$ (**d**) $N_{t}=0.3,\;N_{TC}=1.2,\;N_{b}=0.4,\;b=0.7,\;a=0.3,\;N_{CT}=0.9,\;Re=0.5,\;S_{1}=0.3,\;R_{m}=0.5,\;G_{rc}=1.4,\;x=0.6,\;G_{rt}=0.5,\;G_{rF}=0.8,$ $\alpha =0.8,\;Wi=0.3,\;\tilde{\eta }=1,\;\tilde{\mu }=1$.

**Figure 7:**
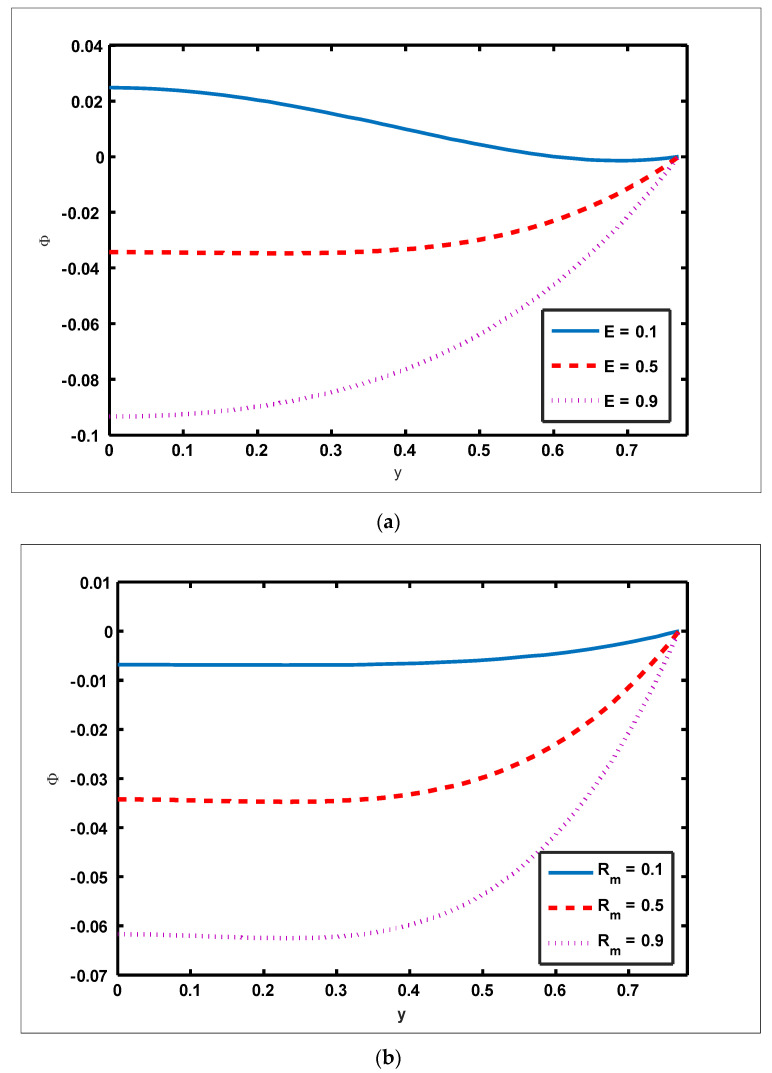
Magnetic force function for induced electric field and magnetic Reynolds number. Values of other physical parameters are (**a**) $N_{t}=0.3,\;N_{TC}=1.2,\;N_{b}=0.4,\;b=0.7,\;a=0.3,\;N_{CT}=0.9,\;R_{m}=0.5,\;G_{rc}=1.4,\;x=0.6,\;G_{rt}=0.5,\;G_{rF}=0.8,$ $\alpha =0.8,\;Q=3,\;\tilde{\eta }=1,\;\tilde{\mu }=1.$ (**b**) $N_{t}=0.3,\;N_{TC}=1.2,\;N_{b}=0.4,\;b=0.7,\;a=0.3,\;N_{CT}=0.9,\;E=0.5,\;G_{rc}=1.4,\;x=0.6,\;G_{rt}=0.5,\;G_{rF}=0.8,$ $\alpha =0.8,\;Q=3,\;\tilde{\eta }=1,\;\tilde{\mu }=1$.

To highlight the outcomes of temperature on Soret $\left(N_{CT}\right),$ Dufour $\left(N_{TC}\right)$, Brownian motion $\left(N_{b}\right),$ and thermophoresis $\left(N_{T}\right)$ parameters, Figure 1a–d is plotted. It is shown in Figure 1a–d that increases in the Soret, Dufour, Brownian motion, and thermophoresis parameters result in a rise in the temperature profile. It is because temperature has a direct link with the constraints of Soret, Dufour, Brownian motion, and thermophoresis. To observe the impact of concentration on Soret $\left(N_{CT}\right),$ Dufour $\left(N_{TC}\right)$, Brownian motion $\left(N_{b}\right),$ and thermophoresis $\left(N_{t}\right)$ constraints, Figure 2a–d is drawn. It is witnessed in Figure 2a–d that the concentration profile tends to fall as the values of $N_{TC}$, $N_{CT}$, $N_{t},$ and $N_{b}$ parameters increase. This is because spontaneous motion interacts with micro-mixing and haphazard collision behavior of solid nanoparticles, dispersing the solid nanoparticles and lowering the solute concentration. Figure 3a–d depicts nanoparticle reactions for greater values of $N_{TC}$, $N_{CT}$, $N_{t},$ and $N_{b}$. By growing the values of $N_{CT},$ $N_{TC},$ $\mathrm{and}\;N_{t}$, the nanoparticle drops (see Figure 3a–c). However, the adverse effect is noted for the case of Brownian motion $\left(N_{b}\right)$. Here, nanoparticle fraction boosts due to enhancing values of $N_{b}$ (see Figure 3d). The impact of Wi, Re, α, and $G_{rF}$ on pressure rise is demonstrated in Figure 4a–d. It is witnessed in Figure 4a that enhancing Wi causes pressure rise to increase in the peristaltic (Q>0,Δp>0), retrograde (Q<0,Δp>0), and free (Δp=0) pumping zones, but increasing Wi causes pressure rise to increase in the augmented (Q>0,Δp<0) pumping area. However, the adverse effect is noted for the case of Re and α. It is noted that increasing *Re* and α causes pressure rise to diminish in the peristaltic (Q>0,Δp>0), retrograde (Q<0,Δp>0), and free (Δp=0) pumping zones, whereas increasing Re and α causes pressure rise to surge in the augmented (Q>0,Δp<0) pumping zone (see Figure 4b–c). Impact of $G_{rF}$ on pressure rise is explained in Figure 4d. It is found in Figure 4d that pressure rise tends to reduce in all peristaltic zones. The significance of Wi, $N_{t},$ $G_{rF},$ and α on the pressure gradient is demonstrated in Figure 5a–d. It is represented in Figure 5a–d that the pressure gradient is minimal in a wider section of the channel, between x∈[0, 0.6] and x∈[0.9, 1], depicting that flow can pass easily without inducing a larger pressure gradient. The pressure gradient is significant in the narrow section of the channel x∈[0.6, 0.9], indicating that a higher pressure gradient is crucial to sustain the flux. Furthermore, as Wi, $N_{T},$ $G_{rF},$ and α grow, the magnitude of the pressure gradient increases. To illustrate the impact of flow quantities on Wi, $G_{rc},$ $G_{rF}$, and Q, Figure 6a–d is plotted. It is observed in Figure 6a–d that the magnitude value of the velocity profile increases due to increasing behavior of Wi and $G_{rc}$ when y∈[0, 0.3], but the opposite behavior is sustained when y∈[0.3, 0.78]. In this region, the magnitude value of velocity decreases by increasing Wi and $G_{rc}.$ It is noted in Figure 6c that totally opposite effects are sustained by the velocity profiles with an increase in $G_{rF}.$ Here, the magnitude of velocity lessens due to the escalating behavior of $G_{rF}$ when y∈[0, 0.3], but velocity increases due to a reduction in the drag force when y∈[0.3, 0.78]. It is illustrated in Figure 6d that velocity enhances when volume flow rate increases. To study the impact of the magnetic force function, Figure 7a,b is drawn. It is seen from these figures that the magnitude of magnetic force function tends to grow by rising E and $R_{m}$ values.

**Figure 8:**
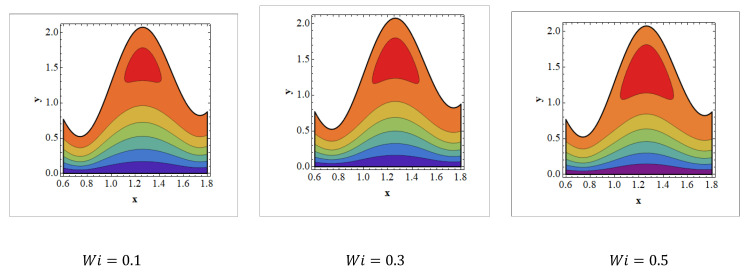
Streamlines for Weissenberg number. Values of other physical parameters are $N_{t}=0.3,\;N_{TC}=1.2,\;N_{b}=0.4,\;b=0.7,\;a=0.3,\;N_{CT}=0.9,\;Re=0.5,\;S_{1}=0.3,\;R_{m}=0.5,\;G_{rc}=1.4,\;G_{rt}=0.5,\;G_{rF}=0.8,$ $\alpha =0.8,\;Q=3,\;\tilde{\eta }=1,\;\tilde{\mu }=1$.

**Figure 9:**
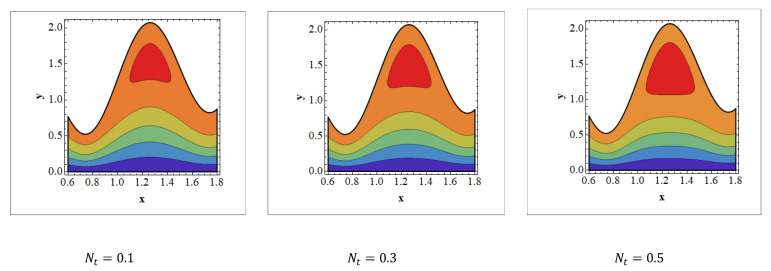
Streamlines for thermophoresis parameters. Values of other physical parameters are $Wi=0.3,\;N_{TC}=1.2,\;N_{b}=0.4,\;b=0.7,\;a=0.3,\;N_{CT}=0.9,\;Re=0.5,\;S_{1}=0.3,R_{m}=0.5,\;G_{rc}=1.4,\;G_{rt}=0.5,\;G_{rF}=0.8,$ $\alpha =0.8,\;Q=3,\;\tilde{\eta }=1,\;\tilde{\mu }=1$.

**Figure 10:**
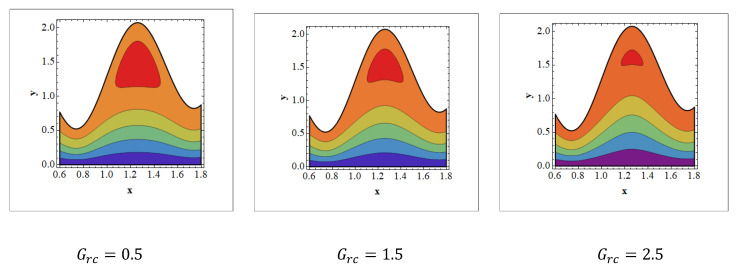
Streamlines for solutal Grashof number. Values of other physical parameters are $N_{t}=0.3,\;N_{TC}=1.2,\;N_{b}=0.4,\;b=0.7,\;a=0.3,\;N_{CT}=0.9,\;Re=0.5,\;S_{1}=0.3,\;R_{m}=0.5,\;Wi=1.4,\;G_{rt}=0.5,\;G_{rF}=0.8,$ $\alpha =0.8,\;Q=3,\;\tilde{\eta }=1,\;\tilde{\mu }=1$.

Trapping is an infrequent occurrence in peristaltic progressed flows. It is stimulated by the internal movement of a fluid mass enclosed by peristaltic wave streamlines. With peristaltic movement at high flow rates and large occlusions, streamlines capture the fluid mass bolus and carry it forward. The aspects of streamlines are depicted in Figure 8, Figure 9 and Figure 10 for various values of $Wi,\;G_{rc},$ and $N_{t}$. It should be noticed in Figure 8 and Figure 9 that when the value of Wi and $N_{t}$ grows, trapped bolus size tends to grow. The opposite behavior is observed Figure 10. It is noted in Figure 10 that when $G_{rc}$ increases, the size of the trapped bolus decreases.

.

## 5. Conclusions

This section describes the overall interpretations of the existing problem. The peristaltic propulsion of a Johnson–Segalman nanofluid under the dependency of a double-diffusion convection and induced magnetic field is the purpose of the current study. Numerical methodology is used to solve nonlinear equations and the graphical results are used to assess various embedded parameters. The main findings are as follows:

- The magnitude of the velocity profile enhances due to increasing behavior of Wi and $G_{rc}$ when y∈[0, 0.3], but the opposite behavior is sustained when y∈[0.3, 0.78].
- The magnitude of the magnetic force function grows as $R_{m}$ and E are enhanced.
- The temperature profile tends to rise and the concentration profile drops as Dufour, Soret, thermophoresis, and Brownian motion constraints are increased.
- The nanoparticle fraction decreases as Dufour, Soret, and thermophoresis parameters are enhanced but an adverse impact is noted for the parameter of Brownian motion.
- The trapped bolus size tends to grow by enhancing the values of Wi and $N_{t}.$

## Data Availability

Not applicable.

## Nomenclature

g	acceleration
$\mu_{e}$	magnetic permeability
E	induced electric field
V	velocity vector
β	relaxation time
α	slip parameter
(W, $W_{1})$	velocity gradient symmetric and skew symmetric part
γ	solutal (species) concentration
Le	Lewis number
Pr	Prandtl number
$N_{TC}$	Dufour parameter
δ	wave number
$D_{CT}$	Soret diffusively
${\left(\rho c\right)}_{p}$	heat capacity of fluid
$\hat{a}$	wave amplitude
c	wave speed
$G_{rt}$	thermal Grashof number
θ	temperature
${\left(\rho c\right)}_{f}$	effective nanoparticle heat capacity
$D_{B}$	Brownian diffusion coefficient
$\beta_{C}$	volumetric solutal expansion coefficient
$\rho_{p}$	nanoparticle mass density
$\rho_{f}$	density of fluid
T	temperature
t	time
σ	electric conductivity
J	current density
$\beta_{T}$	volumetric thermal expansion coefficient
$(\tilde{\mu },$ $\tilde{\eta })$	dynamic viscosities
P	pressure
$D_{TC}$	Dufour diffusively
$N_{b}$	Brownian motion
$N_{t}$	thermophoresis parameter
Ln	nanofluid Lewis number
$N_{CT}$	Soret parameter
Re	Reynolds number
$D_{S}$	solutal diffusively
k	thermal conductivity
${\hat{b}}_{0}$	inlet half-width
$G_{rF}$	Grashof number of nanoparticles
$G_{rc}$	solutal Grashof number
Ω	nanoparticle fraction
$\hat{b}$	half-width of conduit at axial distance
$D_{T}$	thermophoretic diffusion coefficient
C	concentration
$\rho_{f_{0}}$	density of fluid $T_{0}$
Θ	volume fraction nanoparticle
$\left(\frac{d}{dt}\right)$	material derivative
